# Clinical outcomes associated with *Staphylococcus aureus* and *Pseudomonas aeruginosa* airway infections in adult cystic fibrosis patients

**DOI:** 10.1186/s12890-015-0062-7

**Published:** 2015-06-21

**Authors:** Heather G. Ahlgren, Andrea Benedetti, Jennifer S. Landry, Joanie Bernier, Elias Matouk, Danuta Radzioch, Larry C. Lands, Simon Rousseau, Dao Nguyen

**Affiliations:** 1 Department of Medicine, McGill University, Montreal, Canada; 2 Department of Medicine, McGill University Health Center Research Institute, Montreal, Canada; 3 Department of Epidemiology, Biostatistics & Occupational Health, McGill University, Montreal, Canada; 4 Respiratory Epidemiology and Clinical Research Unit, McGill University Health Center, Montreal, Canada; 5 Adult CF clinic, McGill University Health Center, Montreal, Canada; 6 Department of Human Genetics, McGill University, Montreal, Canada; 7 Department of Pediatrics, McGill University Health Center Research Institute, Montreal, Canada

**Keywords:** Cystic fibrosis, Microbiology, *Staphylococcus aureus*, *Pseudomonas aeruginosa*, Lung function, Pulmonary exacerbation, Retrospective cohort

## Abstract

**Background:**

*Staphylococcus aureus* (SA) is the most prevalent organism infecting the respiratory tract of CF children, and remains the second most prevalent organism in CF adults. During early childhood, SA infections are associated with pulmonary inflammation and decline in FEV_1_, but their clinical significance in adult CF patients is poorly characterized.

**Methods:**

We conducted a retrospective cross-sectional study to determine the association between airway microbiology and clinical outcomes (FEV_1_, rate of pulmonary exacerbations, CRP levels and clinical scores).

**Results:**

In a cohort of 84 adult CF patients, 24 % were infected with SA only, 60 % were infected with PA, and 16 % had neither PA nor SA. CF patients with SA experienced fewer pulmonary exacerbations and lower CRP levels than those with PA.

**Conclusion:**

In adult CF patients, SA infections alone, in the absence of PA, are a marker of milder disease.

**Electronic supplementary material:**

The online version of this article (doi:10.1186/s12890-015-0062-7) contains supplementary material, which is available to authorized users.

## Background

Pulmonary disease is the primary cause of morbidity and mortality in patients with cystic fibrosis (CF). Most patients experience acute symptoms during episodes of pulmonary exacerbations, and develop progressive lung disease caused by both chronic airway infections and host inflammation [1]. While it is increasingly recognised that CF airway infections are polymicrobial, *Pseudomonas aeruginosa* (PA) and *Staphylococcus aureus* (SA) are the most prevalent organisms [2, 3]. PA, the dominant airway pathogen, chronically infects up to 60–75 % of adult CF patients [4], and is strongly associated with inflammation, decline in lung function and increased mortality [1, 5–7]. SA, on the other hand, is the most prevalent organism during childhood, and often the first one isolated in CF children [4, 8].

The overall prevalence of SA infections has increased over time, both with methicillin sensitive (MSSA) and methicillin resistant (MRSA) SA [4, 9]. Several studies have examined the clinical impact of SA infections, but all have focused on infants and children [10–14]. In the pre-antibiotic era, SA was a major cause of death in CF children. In more recent studies, SA infection in young children is associated with a decline in lung function [11, 12], as well as increased markers of pulmonary inflammation [15, 16]. Consequently, antibiotic therapy to eradicate SA is used in some centers, but its routine use remains controversial [10, 17–20]. As patients transition from adolescence into adulthood, the prevalence of SA decreases gradually but remains significant, with over 40 % of adults harbouring SA [4]. In older children, SA infections are not consistently associated with poor prognosis, but perhaps even better survival post-transplantation, as SA infection prior to transplantation is an independent factor associated with improved survival [21, 22]. The clinical significance of SA in adult CF patients remains unknown.

We carried out a retrospective cross-sectional study to examine the association between airway infection (SA alone, PA, or neither) and lung function (FEV_1_ % predicted) as well as pulmonary exacerbation rate. We also examined several secondary outcomes, namely plasma C-reactive protein (CRP) and clinical scores.

## Methods

### Study design

This retrospective cross-sectional cohort study included all patients followed at the Montreal Chest Institute (McGill University Health Centre, QC, Canada) Adult CF clinic who were alive in January 2012. Inclusion criteria were as follows: age >18 years old; diagnosis of CF; at least one sputum microbiology culture; at least one routine visit between January 2011 and February 2013. The baseline visit was defined as the first routine visit during which the patient was in a stable clinical condition within the study period. A stable clinical condition was defined as a period of at least 28 days without intravenous (IV) or oral antibiotics (excluding chronic antibiotic treatment). Patients were excluded if they had no routine visit during our study period (*n* = 6) or were colonized with *Burkholderia cepacia* complex (*n* = 1). All study participants provided written informed consent. The study was approved by the Research Ethics Board of the McGill University Health Center.

### Demographic and clinical data

The demographic and clinical data were collected from the CF clinic database, a prospective database of all clinic patients and visits. Demographic data included age and sex. The body mass index (BMI) was calculated from the weight and height. Spirometry, including forced expiratory volume in 1 s (FEV_1_) and forced vital capacity (FVC) expressed as a percentage of predicted values, was performed according to the American Thoracic Society standards [23] and was measured at the baseline visit for this study. Pulmonary exacerbations are episodes of clinical deterioration diagnosed clinically by the treating physician, defined as episodes requiring IV antibiotic treatment at home or in the hospital. The exacerbation rate was calculated using the number of pulmonary exacerbations during the calendar year.

We used the Matouk clinical score, a previously described and validated measure of CF disease activity [24, 25]. The total clinical score includes key manifestations of CF disease with clinical (signs and symptoms), radiographic (chest radiographic findings), pulmonary function and complication subscores as previously described [24, 25]. For this study, we excluded the microbiology subscore (originally described in [24, 25]) to avoid a collinearity effect between the total clinical score and the airway infection status. The total clinical score (out of total of 95 points) is scaled to indicate more severe diseases with lower scores.

### Microbiology and laboratory data

All spontaneously expectorated sputum samples collected from patients, during both routine and non-routine visits as well as hospitalisations, were analyzed by the McGill University Health Centre clinical microbiology laboratory according to standard protocols for CF samples. Briefly, sputum samples were cultured on sheep blood agar, MacConkey agar, and *B. cepacia* agar plates (BD), and the presence of PA, SA, and *B. cepacia* were confirmed through standard biochemical testing. The patients’ infection status was determined based on the microbiology results of all sputum cultures collected during the calendar year of the baseline visit, including both routine and exacerbation visits. Patients were defined as infected with PA and/or SA if ≥50 % of their sputum samples were positive. In the case of insufficient samples, sputum microbiology results from the previous calendar year were used. No differentiation was made between MSSA and MRSA infections in our primary analyses because of the small number of patients with MRSA in our clinic (*n* = 4). Plasma C-reactive protein (CRP) levels were measured from plasma samples collected during the baseline visit using ELISA.

### Statistical analysis

Continuous variables (age, body mass index, FEV_1_ and FVC % predicted, clinical score and subscores, and CRP) were compared among the different infection groups using one-way analysis of variance (ANOVA) or student’s t-test. Post-hoc analysis was done in conjunction with ANOVA using Tukey’s test. Categorical variables (sex) were compared using the Mantel-Haenszel chi-square test. Differences in exacerbation rate and hospitalization rate were calculated using Poisson regression. *P-*values of ≤0.05 were considered statistically significant. To assess the association between airway infection and the patient’s clinical status, we used FEV_1_ % predicted and exacerbation rate as primary outcomes, and plasma CRP and clinical scores as secondary outcomes. Multivariable analyses included variables (age and sex) determined *a priori*. Linear regression was used for continuous outcomes (FEV_1_ % predicted, plasma CRP and clinical scores), and Poisson regression was used for count data outcomes (exacerbation rate). Variables were tested for collinearity using a correlation matrix. In multivariable analyses, the infection category “PA” was used as a reference group, as this was the largest group. Statistical analyses were conducted using SAS 9.2 statistical package (SAS Institute Inc., USA).

## Results

A total of 91 adult patients were followed at the Montreal Chest Institute CF clinic during the study period, and 84 patients were included in this study. We analyzed the microbiology of a total of 366 sputum samples collected during the study period (average 4.3 samples per patient): 37 % of samples were positive for SA (including *n* = 4 for MRSA), and 70 % were positive for PA. The study patients were classified into one of 3 groups based on their PA and SA airway infection status, namely “PA” (*n* = 50; 59 %), “SA only” (*n* = 20; 24 %), and “no PA/SA” (*n* = 14; 17 %) for patients infected with neither PA nor SA. The patients’ demographic and clinical outcomes for each group are presented in Table 1. The identification of microorganisms other than PA and SA, namely *Aspergillus* and *Stenotrophomonas maltophilia*, in each of the 3 groups are summarized in Additional file 1: Table S1. The patients in the different infection groups were similar in age, sex and BMI. There was a trend suggesting that “PA” patients were associated with worse FEV_1_ and FVC % than “SA only” and “no PA/SA” patients. In univariate analyses, the infection groups significantly differed in their exacerbation rate, clinical scores and CRP levels. Across these clinical outcomes, “SA only” patients were similar to “no PA/SA” patients, while “PA” patients experienced the highest rate of exacerbation, CRP and lowest clinical score.

**Table 1:** Clinical characteristics of cohort patients based on infection status

	No PA/SA	SA only	PA	*P-value*
n (%)	14 (16.7 %)	20 (24.1 %)	50 (59.5 %)	-
Age (years)	30.9 (±13.7)	28.5 (±12.2)	35.6 (±12.8)	0.097
Female sex (%)	8 (57.1 %)	9 (45.0 %)	25 (50.0 %)	-
Number of sputum samples	3.6 (±3.7)	3.3 (±2.5)	5.5 (±4.3)	0.055
Number of routine visits	6.6 (±5.3)	4.4 (±3.6)	7.9 (±6.9)	0.096
BMI	21.1 (±2.5)	22.5 (±3.5)	22.0 (±4.5)	0.608
FEV_1_ (% predicted)	70.9 (±21.0)	66.5 (±22.1)	56.3 (±27.2)	0.096
FVC (% predicted)	85.8 (±22.1)	82.8 (±19.3)	72.7 (±26.3)	0.110
Exacerbation rate	0.43	0.20	1.20	<0.02*
Hospitalization rate	0.21	0.15	1.30	<0.10
Total clinical score (out of 95)	71.1 (±14.1)	68.6 (±10.9)	60.8 (±14.6)	0.017^a^
Clinical subscore (out of 45)	35.3 (±4.6)	35.1 (±3.3)	33.0 (±3.8)	0.048*
Radiographic subscore (out of 25)	18.9 (±4.0)	17.7 (±2.9)	15.9 (±2.7)	0.003^a^
Pulmonary function subscore (out of 25)	18.5 (±5.1)	18.0 (±5.1)	15.3 (±6.2)	0.081
Complication subscore (out of 37)	1.5 (±2.3)	2.3 (±2.6)	3.4 (±4.2)	0.185
CRP^†^	2.50 (±3.5)	4.8 (±3.2)	10.6 (±10.0)	0.005^a,b^

*SA* = *Staphylococcus aureus*; *PA* = *Pseudomonas aeruginosa*; *SD* = standard deviation; *BMI* = body mass index; *FEV*_1_ = forced expiratory volume in 1 s; *FVC* = forced vital capacity; *CRP* = C-reactive protein

Data are expressed as mean (± SD) or number (% total), except for the exacerbation and hospitalization rates expressed as mean rates calculated by Poisson regression

*P-*values were determined using the ANOVA or Chi-squared test. For exacerbation rate and hospitalization rate, *P-*values were determined by a comparison with a null model

Comparisons between groups were done using Tukey’s test. Tukey’s test reported the following differences: ^a^*p* < 0.05, “No PA/SA” vs. “PA”

^b^*p* < 0.05, “SA only” vs. “PA”

^†^CRP data was only available in a subset of patients and results in Table 2 were collected from the different groups are as follows: Group No PA/SA: 11/14; Group SA only: 16/20; Group PA: 35/50

To adjust for confounding factors that affect CF disease such the age and sex, we next performed multivariable analyses. Although there was a trend suggesting that “no PA/SA” patients had better FEV_1_ compared to “PA” patients using a linear regression model, we found no statistically significant association between airway infection status and FEV_1_, (Table 2). Next, we compared the exacerbation rate of the different airway infection groups using a Poisson regression model, as presented in Table 3. Compared to “PA” patients, both “SA only” and those with “no PA/SA” were associated with a significantly lower rate of pulmonary exacerbation. In the adjusted model, “SA only” patients were associated with a relative risk of 0.2 (95 % CI [0.1-0.4]) compared to “PA” patients. We also examined several secondary outcomes, namely CRP (Table 4) and the modified Matouk total clinical score (Table 5), a clinical subscore (which quantifies signs, symptoms and complications of CF disease). The “SA only” patients were associated with reduced CRP levels (in both unadjusted and adjusted models) and higher clinical scores (unadjusted model only) compared to “PA” patients. Interestingly, patients with “no PA/SA” showed similar results to “SA only” patients. Taken together, these analyses suggest that “SA only” patients overall have significantly better clinical outcomes than “PA” patients.

**Table 2:** Association between infection status and FEV_1_ %

	Crude			Adjusted		
	Regression	95 % CI	*P*-value	Regression	95 % CI	*P*-value
	Coefficient			Coefficient		
No PA/SA	14.6	−0.4 to 29.5	0.059	14.0	−1.2 to 29.3	0.074
SA only	10.2	−2.9 to 23.2	0.131	9.2	−4.4 to 22.8	0.187
PA	0	Reference	-	0	Reference	-

Regression coefficients were calculated using a linear regression. The adjusted model includes age and sex as covariates

**Table 3:** Association between infection status and exacerbation rate

	Crude Risk Ratio	95 % CI	*P-*value	Adjusted Risk Ratio	95 % CI	*P-*value
No PA/SA	0.4	0.4 to 0.8	0.016	0.3	0.1 to 0.8	0.011
SA only	0.2	0.1 to 0.5	0.0005	0.2	0.1 to 0.4	0.0004
PA	1.0	Reference	-	1.0	Reference	-

Risk ratios were calculated using a Poisson regression. The adjusted model includes age and sex as covariates

**Table 4:** Association between infection status and C-reactive protein

	Crude			Adjusted		
	Regression	95 % CI	*P*-value	Regression	95 % CI	*P*-value
	Coefficient			Coefficient		
No PA/SA	−8.0	−13.4 to −2.7	0.005	−8.1	−13.5 to −2.6	0.005
SA only	−5.8	−10.5 to −1.1	0.019	−5.8	−10.8 to −0.8	0.026
PA	0	Reference	-	0	Reference	-

Regression coefficients were calculated using a linear regression. The adjusted model includes age and sex as covariates

**Table 5:** Association between infection status and total clinical score

	Crude			Adjusted		
	Regression	95 % CI	*P*-value	Regression	95 % CI	*P*-value
	Coefficient			Coefficient		
No PA/SA	10.3	2.1 to 18.4	0.016	9.9	1.6 to 18.1	0.022
SA only	7.8	0.7 to 14.9	0.035	7.0	−0.4 to 14.4	0.068
PA	0	Reference	-	0	Reference	-

Total clinical score is marked out of 95 points. Regression coefficients were calculated using a linear regression. The adjusted model includes age and sex as covariates

In all four crude models, “no PA/SA” and “SA only” groups had better total clinical scores and subscores than the reference “PA only” group. These trends were also observed in the adjusted models, although only the comparison between “no PA/SA” and “PA only” groups remained statistically significant. Consistent with the observation that patients infected with “no PA/SA” and “SA only” had less active disease than those infected with “PA only”, their plasma CRP levels were lower in both crude and adjusted models.

Among PA infected patients in the “PA” group, 10 out of 50 were co-infected with both PA and SA. The demographic and clinical characteristics of patients based on their infection group, with the “PA only” and “PA + SA” groups separated, are presented in the Additional file 1: Table S2, and both groups showed no significant differences (Table 6). In order to determine whether including the “PA + SA” patients to the “PA” group had a significant effect on our results, we excluded the “PA + SA” patients and repeated the univariate and multivariate analyses. As shown in the Additional file 1: Table S3 to S6, all results were similar. Furthermore, 4 out of 84 patients (4.8 %) in our cohort were MRSA, and all were co-infected with both PA and SA. The results of all univariate and multivariate analyses remained unchanged after exclusion of the MRSA patients (data not shown).

**Table 6:** Clinical characteristics of cohort patients based on infection status ("no PA/SA" excluded)

	PA + SA	PA only	P-value*
Number (%)	10	40	-
Age (years)	30.6 (±9.1)	36.8 (±13.4)	0.175
Female sex (%)	9 (45.0 %)	20 (50 %)	-
BMI	22.1 (±3.4)	21.9 (±4.8)	0.922
FEV_1_ (% predicted)	52.9 (±20.6)	57.1 (±28.8)	0.666
FVC (% predicted)	69.3 (±20.8)	73.6 (±27.7)	0.649
Exacerbation rate	0.9	1.3	<0.9
Hospitalization rate	0.9	0.5	<0.9
Total clinical score (out of 95)	62.3 (±13.6)	60.4 (±15.0)	0.720
Clinical subscore (out of 45)	33.9 (±3.5)	32.8 (±3.9)	0.430
Radiographic subscore (out of 25)	15.4 (±2.5)	16.0 (±2.8)	0.518
Pulmonary function subscore (out of 25)	14.9 (±5.6)	15.4 (±6.4)	0.821
Complication subscore (out of 37)	1.9 (±3.8)	3.8 (±4.3)	0.217
CRP	11.1 (±8.5)	10.4 (±10.5)	0.870

## Discussion

To date, studies reporting on SA infections have exclusively focused on CF children where the prevalence of SA infections is higher. In children under 12 months, 27 % of bronchoalveolar lavage fluids are positive for SA [26]. The prevalence of SA increases during childhood and is highest in children 6 to 10 years old [4]. In young children under the age of 2, SA infections are associated with a decline in lung function [11, 12, 15, 27, 28]. In those under 7, SA infections are associated with pulmonary inflammation, increased neutrophil counts, elevated IL-8, and free neutrophil elastase on BAL [15]. Consequently, antibiotic therapies can be used to prevent or eradicate SA [29–31], but their routine use remains controversial [10, 17–19].

In contrast to children, the clinical significance of SA infections in adult is not established. The presence of SA infections may in fact be a marker of less severe disease, as it could represent the absence of PA. Reports that SA infections in older children are associated with better 5-year survival following transplantation suggest that this may be the case [21, 22]. In our cohort of adult CF patients, 24 % of patients are colonized with SA only, without PA. Patients with SA were associated with lower rates of pulmonary exacerbations and CRP than those with “PA”, suggesting that infection with SA is a marker of less severe disease. Interestingly, we did not identify any significant association between the airway infection status and lung function (FEV_1_ and FVC). Although FEV_1_ is the most commonly used clinical outcome in studies of CF lung disease due to its association with mortality and quality of life [32–35], it may not be as sensitive to the disease activity as the rate of pulmonary exacerbations and clinical scores, particularly in light of our small sample size for the “no PA/SA” and “SA only” groups.

It is also worth noting that among those infected with PA, 20 % (*n* = 10) were co-infected with both PA and SA. In children, several studies have reported that PA + SA co-infections are associated with diminished survival, greater decline in lung function and increased airway inflammation in CF children [11, 15, 16]. Unfortunately, the small size of this group in our study precluded any meaningful subgroup analyses. However, we compared patients with only PA and those with PA + SA and found no significant differences in demographic or clinical parameters. Our findings also did not differ when the PA + SA patients were excluded from our analyses.

In the face of the global rise in MRSA, the prevalence of MRSA has also increased in many CF clinics [36, 37]. Methicillin-resistant SA (MRSA) is present in 4.1 % of CF patients in Canada [38] and has been associated with faster decline in lung function than methicillin-susceptible SA (MSSA) [39]. It has also been shown to be a risk factor for failure to recover from exacerbation [40], and persistent infection has been linked to increased mortality [39]. The prevalence of MRSA in our cohort was 4.8 % and the small number of MRSA+ patients precluded any subgroup analyses. However, our results were unchanged when we performed the analysis of our cohort excluding the MRSA patients. Finally, our study and others likely underestimate the true prevalence of SA infection. Standard clinical laboratories typically do not detect small colony variant SA strains that are slow growing [41] which may be associated with a greater decline in FEV_1_ in CF children [42] and more severe lung disease [43].

## Conclusion

Our study suggests that, in contrast to children, the presence of SA in CF adults, in the absence of PA, is a marker of milder lung disease.

## Additional file

Additional file 1: Table S1. Sputum microbiology for other microorganisms in sputum cultures

## Abbreviations

BMI: Body mass index
CF: Cystic fibrosis
CRP: C-reactive protein
FEV1: Forced expiratory volume in 1 second
MSSA: Methicillin sensitive *Staphylococcus aureus*
MRSA: Methicillin resistant *Staphylococcus aureus*
PA: *Pseudomonas aeruginosa*
SA: *Staphylococcus aureus*
