# Supplementary material for: Clinical outcomes associated with Staphylococcus aureus and Pseudomonas aeruginosa airway infections in adult cystic fibrosis patients
Source: BMC Pulm Med. 2015 Jun 21;15:67. doi: 10.1186/s12890-015-0062-7 (PMC4475617; doi:10.1186/s12890-015-0062-7)
Supplement: Additional file 1: Table S1. — Sputum microbiology for other microorganisms in sputum cultures [file 12890_2015_62_MOESM1_ESM.pdf]

Supplemental information

Table S1. Sputum microbiology for other microorganisms in sputum cultures

	No PA/SA	SA only	PA
Stenotrophomonas maltophilia	1 (7%)	2 (10%)	4(8%)
Aspergillus species	5 (36%)	4 (20%)	12 (24%)

Values expressed in n (%) of patients with at least one positive culture for *Aspergillus* species or S*tenotrophomonas maltophilia*.

Table S2. Clinical characteristics of patients according to their airway infection status

	No PA/SA	SA only	PA+SA	PA only	*P-value* *
n (%)	14 (16.7%)	20 (24.1%)	10 (12.0%)	40 (48.2%)	-
Age (years)	30.9 (±13.7)	28.5 (±12.2)	30.5 (±9.1)	36.8 (±13.4)	0.09
Female sex (%)	8 (57.1%)	9 (45.0%)	5 (50.0%)	20 (50.0%)	-
BMI	21.1 (±2.5)	22.5 (±3.5)	22.1 (±3.4)	21.9 (±4.7)	0.80
FEV_1 (% predicted)	70.9 (±21.0)	66.5 (±22.1)	52.9 (±20.6)	57.1 (±28.8)	0.18
FVC (% predicted)	85.8 (±22.1)	82.8 (±19.3)	69.3 (±20.8)	73.6 (±27.7)	0.20
**Exacerbation rate**	0.43	0.20	0.90	1.27	**<0.01**
Hospitalization rate	0.21	0.15	0.90	0.5	<0.15
**Total clinical score (out of 95)**	71.1 (±14.1)	68.6 (±10.9)	62.3 (±13.6)	60.4 (±15.0)	**0.04**
Clinical subscore (out of 45)	35.3 (±4.6)	35.1 (±3.3)	33.9 (±3.5)	32.8 (±3.9)	0.08
**Radiographic subscore** (out of 25)	18.9 (±4.0)	17.7 (±2.9)	15.4 (±2.5)	16.0 (±2.8)	**<0.01**[1]
Pulmonary function subscore (out of 25)	18.5 (±5.1)	18.0 (±5.1)	14.9 (±5.6)	15.4 (±6.4)	0.17
Complication subscore (out of 37)	1.5 (±2.3)	2.3 (±2.6)	1.9 (±3.8)	3.8 (±4.3)	0.14
**CRP**[†]	2.50 (±3.5)	4.8 (±3.2)	11.1 (±8.5)	10.4 (±10.5)	**0.01**[2]

SA = *Staphylococcus aureus*; PA = *Pseudomonas aeruginosa*; SD = standard deviation; BMI = body mass index; FEV_1 = forced expiratory volume in 1 second; FVC = forced vital capacity; CRP = C-reactive protein.

Values expressed in: n (%) or mean (± SD), except for the exacerbation and hospitalization rates expressed as mean rates calculated by Poisson regression.

* P-values were determined using the ANOVA or Chi-squared test. For exacerbation rate and hospitalization rate, P-values were determined by a comparison with a null model.

Comparisons between groups were done using Tukey's test. Tukey's test reported the following differences: [1] $p<0.05$, "No PA/SA" vs. "PA+SA", "No PA/SA" vs. "PA only"

[2] $p<0.05$, "No PA/SA" vs. "PA only"

† CRP data was not available for all patients and results in Table 2 were collected from the different groups are as follows: Group 1: 11/14; Group 2: 16/20; Group 3: 7/10; Group 4: 28/40.

Table S3. Association between infection status and FEV_1 % (PA+SA excluded)

	Crude Regression Coefficient	95% CI	P-value	Adjusted Regression Coefficient	95% CI	P-value
No PA/SA	13.7	-2.0 to 29.5	0.091	13.4	-2.7 to 29.6	0.108
SA only	9.3	-4.5 to 23.2	0.192	8.4	-6.2 to 23.0	0.263
PA	0	Reference	-	0	Reference	-

Regression coefficients were calculated using a linear regression. The adjusted model includes age and sex as covariates.

Table S4. Association between infection status and exacerbation rate (PA+SA excluded)

	Crude Risk Ratio	95% CI	P-value	Adjusted Risk Ratio	95% CI	P-value
No PA/SA	**0.3**	**0.1 to 0.8**	**0.012**	**0.3**	**0.1 to 0.7**	**0.004**
SA only	**0.2**	**0.1 to 0.4**	**0.0004**	**0.1**	**0.05 to 0.4**	**0.0001**
PA	1.0	Reference	-	1.0	Reference	-

Risk ratios were calculated using a Poisson regression. The adjusted model includes age and sex as covariates.

Table S5. Association between infection status and C-reactive protein (PA+SA excluded)

	Crude			Adjusted		
	Regression Coefficient	95% CI	P-value	Regression Coefficient	95% CI	P-value
No PA/SA	**-7.9**	**-13.4 to -2.4**	**0.007**	**-7.9**	**-13.6 to -2.3**	**0.008**
SA only	**-5.6**	**-10.5 to -0.8**	**0.028**	**-5.5**	**-10.7 to -0.3**	**0.045**
PA	0	Reference	-	0	Reference	-

Regression coefficients were calculated using a linear regression. The adjusted model includes age and sex as covariates.

Table S6. Association between infection status and total clinical score (PA+SA excluded)

	Crude			Adjusted		
	Regression Coefficient	95% CI	P-value	Regression Coefficient	95% CI	P-value
No PA/SA	**10.6**	**2.2 to 19.1**	**0.016**	**10.6**	**2.0 to 19.2**	**0.018**
SA only	**8.2**	**0.7 to 15.6**	**0.034**	7.6	-0.1 to 15.4	0.058
PA	0	Reference	-	0	Reference	-

Total clinical score is marked out of 95 points. Regression coefficients were calculated using a linear regression. The adjusted model includes age and sex as covariates.
